# Social Support and Family Functioning in Chinese Families of Children with Autism Spectrum Disorder

**DOI:** 10.3390/ijerph18073504

**Published:** 2021-03-28

**Authors:** Xianmei Lei, Jiří Kantor

**Affiliations:** 1 Faculty of Special Education, Leshan Normal University, Leshan 614000, China; 201321010110@mail.bnu.edu.cn; 2 Center of Evidence-based Education & Arts Therapies, Faculty of Education, Palacky University, 79900 Olomouc, Czech Republic; 3 Institute of Special Education Studies, Faculty of Education, Palacky University, 79900 Olomouc, Czech Republic

**Keywords:** autism spectrum disorder, caregivers, families, family functioning, social support

## Abstract

Families caring for children with autism spectrum disorder (ASD) have reported poorer family functioning. Social support might strengthen family functioning, but limited research to date has focused on this association in China. This study conducted a cross-sectional survey of Chinese families that have children with ASD to examine the relationship between social support and family functioning. Caregivers of children with ASD from Sichuan province in China (*N* = 167) were surveyed concerning their perceived social support and family functioning. The Social Support Rating Scale was used to investigate caregivers’ perceived social support from three dimensions: subjective support, objective support, and the utilization of support. A Chinese version of the Family Adaptability and Cohesion Scale was used to investigate their perceptions of family cohesion and adaptability. The Pearson correlation coefficient and stepwise multiple regression were used for analyses. The results suggested that social support was positively related to family cohesion and adaptability. Of the three sub-domains of social support, both subjective support and the utilization of support were positively associated with family cohesion and adaptability. The study’s findings evidenced the importance of different types of social support and could be used to develop a targeted support service for families that have children with ASD to improve their family functioning and sustain the family unit.

## 1. Introduction

The population survey from the Centers for Disease Control and Prevention in America reported that about 1 in 54 children are diagnosed with autism spectrum disorder (ASD) [1]. Additionally, recent research also found a high prevalence of autism in China, at around 1% [2], which means an increasing number of parents were faced with the challenges that accompany the diagnosis of ASD [3]. The essential features of ASD are persistent impairment in reciprocal social communication and social interaction, and restricted, repetitive patterns of behavior, interests, or activities [4]. As such, children with ASD face developmental challenges that can significantly affect parental and family functioning [5].

Family functioning is the way in which family members interact with, react to, and treat other family members [6]. It is an important source of support for the family [7]. There has been an increased interest in exploring family functioning in families who raise children with ASD. Olson et al. [8] identified two dimensions that contribute to the overcoming of a stressful event: cohesion and adaptability. Cohesion refers to the emotional connection among family members, while adaptability indicates the ability to change the family structure based on events that occur during the life cycle [9]. Family cohesion was found to be associated with lower maternal depressive symptoms [3] and to positively predict parents’ well-being and quality of life [10,11,12]. Families identified as high on family cohesion (i.e., enmeshed) could implement more positive coping strategies [13]. A recent study suggested family cohesion and adaptability completely mediated the relationship between social support and caregivers’ satisfaction on family quality of life [14]. In a study of parents of preschool children, Wang et al. [15] reported family cohesion and adaptability had effects on children’s mental development. These works reflect the importance of family functioning for both parents and their children.

However, the levels of family cohesion and adaptability in families with children with ASD are at risk of falling outside the healthy family functioning range [16]. The reason is that families of children with ASD have been found to perceive less family adaptability and cohesion than families of typically developing children [16,17]. The characteristics of children with ASD place stress on families with a child with ASD and create conflict within the family system. In Asia, Xue et al. [18] surveyed 65 Singaporean parents of children with ASD and found a lower mean cohesion and adaptability level compared to Taiwanese parents of children with ASD. Zhou [19] studied 166 Chinese parents of children with ASD and reported lower levels of family cohesion and adaptability compared to parents of typically developing children. Lin et al. [20] found that Taiwanese mothers reported significantly lower levels of family adaptability and cohesion than mothers in the U.S. did, and they explained the differences could be related to the different cultures. Poorer functioning in families predicts poorer levels of functioning in the child with ASD [21], thus exploring the factors contributing to successful family functioning is essential.

Social support might have a strong impact on family functioning because many families of individuals with ASD begin by seeking social support and resources to cope with stressful situations while facing challenges and caregiver burden [13,20,22,23]. There is a growing body of evidence to suggest that families that have a child with ASD exhibited healthier adaptation when they perceived adequate social support [13,24,25]. For example, Ekas et al. [3] examined mothers of children with ASD and found greater social support contributed to increased family cohesion. In this case, higher levels of social support were associated with more positive states of mind, which may translate to more emotional bonding within the family system. In another study of parents of children with ASD, Manning et al. [26] found that coping through relying on family and friends positively impacted family functioning. Sawyer et al. [27] reported mothers who perceived more social support had fewer mental health problems because social support helped lighten the parenting burden and then contributed to reducing parental stress. Mercado et al. [28] found more social support predicted lower rates of anxiety and depression in Latinx caregivers who have children with neurodevelopmental disorders. The role of social support was important given that many Latinx caregivers might not have family support. Therefore, social support is considered an important factor related to maternal well-being and family adaptation [20]. In China, families of children with ASD desire social support or resources [29,30], but previous surveys reported that the overall social support for families of children with ASD was limited [31,32]. Additionally, since traditional Chinese culture emphasizes the family over the individual [17], Chinese families’ experiences may be very different from those in the West. Some family members are likely to sacrifice themselves to take care of other family members’ needs. They may get involved in child caring and perceive more stress. As such, families who perceive more social support might have increased family functioning. However, to the best of our knowledge, only one study, Ji et al. [33], examined the relationship between social support and family functioning in Chinese families of children with ASD. Hence, further understanding of the relationship between social support and family functioning in a very different cultural context is necessary.

Social support is a complex construct that comprises many facets [34], and one useful way to conceptualize it is that it has both subjective and objective aspects [34,35]. Xiao [36] divided social support into three constructs according to Chinese environmental and cultural conditions: objective support, subjective support, and the utilization of support. Objective support refers to the visible or actual social support, and subjective support reflects an individual’s level of satisfaction of being supported, while utilization of support is the degree individuals make use of the available social support [36]. Prior studies indicated the roles of different types of social support on family aspects were different. For instance, Leng et al. [37] reported that objective support had a significant correlation with caregivers’ physical health-related quality of life, whereas subjective support and the utilization of support had significantly positive correlations with caregivers’ mental health-related quality of life. Phillips et al. [35] found subjective support was a more important predictor of older persons’ psychological well-being. Similarly, Bristol [38] reported the parents’ belief that their child receiving appropriate services was vital for them to cope with the ongoing stresses related to having a child with ASD. These studies suggested the perception of social support often accounted for more variance on family aspects, such as depression and psychological well-being, than the actual social support [35,39]. Therefore, there is a need to know about the roles of different aspects of social support in influencing caregivers’ family functioning to help develop culturally appropriate interventions for these families. Thus, the current study aimed to assess the relationship between social support and family functioning in a sample of Chinese families of children with ASD. We included three aspects of social support: objective support, subjective support, and the utilization of support. The present study’s findings might help practitioners to develop targeted supports for these families.

## 2. Materials and Methods

A cross-sectional design was used for this research. The study followed the age criteria set by UNESCO that defines children as the period from birth to 18 years old. The participants were caregivers of children with ASD and were recruited through the teachers in special education schools in Sichuan province using a convenience sampling method. The inclusion criteria of the participants were guardians of children under 18 years of age and diagnosed with autism spectrum disorder, volunteers, and able to answer questionnaires. Medical records were used to identify the diagnosis of the child and level of functioning. ASD of parents’ children were not re-diagnosed by the authors. We contacted the caregivers of children with ASD through the teachers of the special education schools. Then, we explained the purpose of the study and the confidentiality of the data to the caregivers. The caregivers who agreed to participate were asked to fill out the questionnaires in pencil and paper. The research was approved by Leshan Normal University and the ethics committee of Palacký University, Faculty of Education (3S/2015).

A total of 167 caregivers for children with ASD in Sichuan Province were selected as participants. Table 1 provides information regarding caregivers’ and children’s characteristics. Participants included 94 mothers (56.30%), 37 fathers (22.20%), 31 grandparents (18.60%), and 5 uncles and aunts (3.00%). Among them, most were married or living with a partner (*N* = 143, 85.60%), many had only a senior high school degree or less (*N* = 108, 64.70%), and nearly half were unemployed (*N* = 76, 45.50%). The proportion of caregivers who reported that they lived in cities was 50.90%, and many had monthly income less than RMB 4000 (about USD 580) (*N* = 111, 66.40%). In terms of child characteristics, there were 112 boys (67.10%) and 54 girls (32.30%), with a mean age of 9.72 years (SD = 2.958), and the majority had moderate or (very) low functional level (*N* = 139, 83.20%). See Table 1 for more details about the research sample.

The measures used in the study included a brief demographic questionnaire, the Social Support Rating Scale, and the Family Adaptability and Cohesion Scale. A brief demographic questionnaire was used to collect information including the child’s age, gender, and severity level, caregiver’s marital status, educational level, employment status, monthly household income, place of residence, and caregiver-child relationship.

The Social Support Rating Scale (SSRS), compiled by Xiao [36] based on Chinese conditions, is a 10-item scale including three dimensions: subjective support (four items), objective support (three items), and the utilization of support (three items). Higher scores indicate more subjective social support, objective social support, and better utilization of social support. The scores of subjective support from 8 to 24 are defined as low, from 25 to 32 as high; the scores of objective support from 1 to 13 are defined as low, from 14 to 22 as high; the scores of the utilization of support from 3 to 9 are defined as low, from 10 to 12 as high [40,41]. The scale has been widely used in the Chinese population and has good reliability and validity [42]. The Cronbach alpha coefficient of the scale for this sample was 0.704.

The Chinese version of the Family Adaptability and Cohesion Scale (FACESⅡ-CV) was used to evaluate family cohesion and adaptability. This 30-item self-report scale was developed by Olson et al. in 1982 and translated by Phillips et al. in 1991. The original scale includes the participant’s perception of actual and ideal family conditions. In the current study, the respondents reflected the actual conditions on the 5-point Likert scale with the poles from “almost never” to “almost always.” Higher scores indicate higher family cohesion and adaptability. Scores for cohesion are described as very low (<55.90), moderately low (55.90–63.90), moderately high (64.00–71.90), and very high (>71.90). Scores for adaptability are also classified as very low (<44.70), moderately low (44.70–50.90), moderately high (51.00–57.10), and very high (>57.10) [8,15]. The Chinese version has been verified with high retest reliability, internal consistency, and convergence validity [43]. In this sample, the Cronbach alpha coefficient for the scale was 0.912.

SPSS version 18.0 software was used for the statistical processing. The reliability of the scale was determined. Descriptive statistics of demographic variables, SSRS, and FACESⅡ-CV were calculated. Pearson’s correlation analysis was used to assess the relationship between social support and family functioning in caregivers of children with ASD. A stepwise multiple regression analysis was conducted to identify the contribution of social support to family functioning. A *p* < 0.05 was considered statistically significant.

## 3. Results

### 3.1. Scores of Family Cohesion and Adaptability

The mean scores of family cohesion and adaptability were 65.80 (SD = 10.34) (moderately high) and 45.61 (SD = 8.82) (moderately low). The mean objective support score was 7.42 (SD = 3.30) (low level), the mean subjective support score was 20.67 (SD = 5.66) (low level), and the mean utilization of support score was 6.69 (SD = 1.95) (low level).

### 3.2. Correlations of Social Support and Family Functioning

As demonstrated in Table 2, positive associations were found between objective support and family cohesion (r = 0.300, *p* < 0.001) and family adaptability (r = 0.206, *p* < 0.01); between subjective support and family cohesion (r = 0.406, *p* < 0.001) and family adaptability (r = 0.402, *p* < 0.001); between the utilization of support and family cohesion (r = 0.366, *p* < 0.001) and family adaptability (r = 0.335, *p* < 0.001). It indicated families with more social support could perceive better family functioning, and vice versa.

### 3.3. Using Multiple Regression Analysis to Identify Factors Predicting Family Functioning

A stepwise multiple regression analysis was conducted with family cohesion and adaptability as dependent variables, respectively, and subjective support, objective support, utilization of support as independent variables, as shown in Table 3. The results indicated that subjective support and the utilization of support remained as predictors of family cohesion and adaptability, respectively accounting for 19.20% (∆R^2^ = 0.192, F = 19.448, *p* < 0.001) and 17.70% (∆R^2^ = 0.177, F = 17.819, *p* < 0.001) of the total variance. Both subjective support and the utilization of support were found to be positively associated with caregivers’ family cohesion (β = 0.296, t = 3.559, *p* < 0.001; β = 0.222, t = 2.665, *p* = 0.009 < 0.01, respectively) and adaptability (β = 0.309, t = 3.675, *p* < 0.001; β = 0.186, t = 2.220, *p* = 0.028 < 0.05, respectively). It indicated that caregivers with higher subjective support and utilization of support had increased family cohesion and adaptability.

## 4. Discussion

This study aimed to explore the relationship between social support and family functioning in Chinese families of children with ASD. Consistent with prior research [33], the current study found that social support was positively related to family functioning. Ji et al. [33] measured social support with the Multidimensional Scale of Perceived Social Support that evaluated caregivers’ perceived social support from family, friends, and significant others. Our findings contributed to the research by differentiating between subjective support, objective support, and the utilization of support when examining the relationship of social support and family functioning among families of children with ASD.

Social support has been consistently identified as one of the most powerful predictors of psychological adjustment [44]. Research indicated that receiving more social support decreased depressed family mood. Similar to Benson’s [44] view that maternal perceptions of the availability and quality of social support are linked to reduced distress among mothers of children with ASD, the current study found that subjective support (i.e., the individuals’ level of satisfaction of being supported) acted as the predictor of family cohesion and adaptability in Chinese caregivers of children with ASD, while objective support (i.e., the visible or actual social support) did not. This finding was also similar to those conducted in America, e.g., [28] and in China with other groups, e.g., [35,40]. It means compared to formal sources of support, caregivers’ beliefs about receiving adequate social support for themselves and their child was more important for successful family adaptation [25]. The current study indicated that the perception of social support was more important than the actual social support [41]. According to Xiao [36], subjective assessment of received social support was more meaningful than objective measures of social support because it is a psychological perception of reality and can affect an individual’s behavior and development. Recent studies showed that perceived social support of parents has multiple benefits for the child with ASD, e.g., on his/her emotions and behavior [45]. This finding is important for all professionals working with families of children with ASD. The family members’ subjective perception is of the highest importance and should be utilized for providing individualized professional services [46]. Future research should focus on exploring lived experiences of families with different types of social support and study what is perceived as helpful and effective by them [47]. The present study provided evidence that perceived social support played a role in family hardiness [48], life satisfaction [49], quality of life [50], family adaptation, and maternal well-being [20,44,51], and it was an integral coping resource in the caregiving stress and adaptation process [52].

However, caregivers were found to perceive a lower level of subjective support in this study. This was in accordance with the finding of Siklos et al. [25] that parents of children with ASD perceived less satisfaction with the support received. It suggested that enhancing subjective social support to caregivers of children with ASD was critical. Even though objective support did not predict caregivers’ family cohesion and adaptability in this study, it is worth noting that objective support was also irreplaceable since it served as the foundation for subjective support and utilization of support [40]. Additionally, it was found to have a significant correlation with caregivers’ positive outcomes such as physical health-related quality of life [37].

Moreover, the utilization of support (i.e., the degree of actual use of the available social support) was another predictor of family cohesion and adaptability in the current study. This finding is supported by previous studies in China and other countries, e.g., [20,37]. Leng et al. [37] studied Chinese caregivers of a family member with serious mental illness and reported a higher utility of support was associated with a better quality of life. Lin et al. [20] examined US mothers of individuals with ASD and found higher levels of family cohesion and adaptability were associated with greater use of problem-focused coping (i.e., an attempt to change or manage the stressful situations, which was related to positive caregiver perceptions). These evidenced the important role social support played in promoting positive adaptation.

The present study suggested caregivers had a lower utility of social support. This supported research that indicated families of children with ASD tend to turn more to family members in place of friends [3,52]. Both parent and child characteristics (e.g., parents’ stress and depression, children’s cognitive limitations and behavior problems) influence the parents’ decision to seek social support [53]. Asians, however, are more reluctant to explicitly ask for support from others compared to Westerners because they are more concerned about the potentially negative relational consequences of such behaviors [54]. Disability in any form is highly stigmatized in Asian cultures, which impacts the access and availability of social support [52]. Importantly, Chinese traditional beliefs that family issues should remain and be resolved within the family [18] might account for lower utilization of social support in this sample, which influences family functioning. It further highlighted the importance of giving attention to the utilization of support as targets of intervention to potentially improve family functioning.

### 4.1. Implications

The present study found that social support was positively associated with family cohesion and adaptability. Further, caregivers with higher subjective support and utilization of support had increased family cohesion and adaptability. Based on this, appropriate and meaningful support tailored to this population should be developed. Such activities should focus on increasing the quantity and quality of available support to foster stronger and more adaptive family functioning [18]. In particular, interventions should target helping Chinese families increase the use of available resources to cope with challenges in their daily life. For instance, public education campaigns should be further developed to raise awareness of ASD and reduce any associated stigma of the condition [11,49]. It is likely families would be more able to successfully utilize available support once they change their perceptions [21]. Moreover, techniques in cognitive and behavioral therapy and social rehabilitation such as cognitive reconstruction, behavioral exposure, and coping skills training to decrease proneness to shame might also help achieve this goal effectively [55,56].

### 4.2. Limitations

There were several limitations of the current study and recommendations for future work that should be recognized. First, the caregivers of children with ASD in this study were recruited through the teachers of special education schools in Sichuan Province (the economically underdeveloped regions), so the results may not entirely represent the experiences of families who did not send their children with ASD to special education schools and the groups in other developed regions of China. Research indicates that caregivers of lower socio-economic status might experience higher rates of family conflicts and disharmony [37]. Therefore, future research should enroll participants from different regions and children outside of special education schools to ensure the generalizability of findings and to further address this issue. Second, this study used self-reports to measure caregivers’ perception of social support and family functioning. Self-report research has some inherent limitations, such as memory bias, reporting bias, common method variance, and social desirability bias [41]. Consequently, there is a need to conduct objective research to further determine this issue. Third, this cross-sectional study only analyzed the research outcomes at a certain point in time [37], which made it impossible to determine causal correlations between social support and family functioning. In line with suggestions to gather longitudinal data to establish causal connections between social environment and mental health outcomes [44], longitudinal research in the future is needed to further confirm the relationship between the two constructs.

## 5. Conclusions

The present study shed new light on the relationship of social support and family functioning in families raising children with ASD by differentiating between subjective support, objective support, and the utilization of support. The results of this study showed the predictors of family functioning in families of children with ASD encompassed subjective support and the utilization of support. The relationship between objective support and family functioning was also evident. The findings pointed to the importance of considering different types of social support and could inform practitioners to target subjective support and the utilization of support to promote family functioning when developing socially and culturally appropriate intervention for this group.

## Figures and Tables

**Table 1:** Sample characteristics of the children and caregivers.

Variables	Category	Frequency	%
Characteristics of caregivers			
Marital status	Married or living with a partner	143	85.60
	Divorced, separated, or widowed	23	13.80
	Missing	1	0.60
Educational level	Primary school or less	48	28.70
	Junior school	32	19.20
	Senior high school	28	16.80
	Junior college	27	16.20
	Bachelor degree or above	32	19.20
Employment status	Full-time job	61	36.50
	Part-time job	16	9.60
	Job-waiting	13	7.80
	Unemployment	76	45.50
	Missing	1	0.60
Caregiver–child relationship	Mother	94	56.30
	Father	37	22.20
	Grandparents or others	36	21.60
Place of residence	City	85	50.90
	Town	34	20.40
	Village	48	28.70
Monthly income	≤RMB 2000	46	27.50
	RMB 2001–4000	65	38.90
	RMB 4001–6000	18	10.80
	RMB 6001–8000	14	8.40
	RMB 8001–10,000	13	7.80
	≥RMB 10,000	11	6.60
Characteristics of children			
Age (years)		M = 9.72, SD = 2.958	
Gender	Male	112	67.10
	Female	54	32.30
	Missing	1	0.60
Functional level	High	27	16.20
	Moderate	61	36.50
	Low	66	39.50
	Very low	12	7.20
	Missing	1	0.60

**Table 2:** Correlations analysis of social support and family functioning.

Variables	1	2	3	4	5
1. Family cohesion	1				
2. Family adaptability	0.766 ***	1			
3. Objective support	0.300 ***	0.206 **	1		
4. Subjective support	0.406 ***	0.402 ***	0.371 ***	1	
5. Utilization of support	0.366 ***	0.335 ***	0.393 ***	0.488 ***	1

Note. ** *p* < 0.01, *** *p* < 0.001.

**Table 3:** Multiple regression analysis for social support predicting family functioning.

Dependent Variable	Independent Variables	B	β	T	*p*	R^2^	∆R^2^	F	*p*
Family cohesion	(Constant)	46.787		14.986	<0.001	0.203	0.192	19.448	<0.001
	Subjective support	0.536	0.296	3.559	<0.001				
	Utilization of support	1.178	0.222	2.665	0.009				
Family adaptability	(Constant)	30.062		11.229	<0.001	0.188	0.177	17.819	<0.001
	Subjective support	0.477	0.309	3.675	<0.001				
	Utilization of support	0.843	0.186	2.220	0.028				
